# A Perspective on the Rise of Optofluidics and the Future

**DOI:** 10.3390/mi8050152

**Published:** 2017-05-08

**Authors:** Chaolong Song, Say Hwa Tan

**Affiliations:** 1 School of Mechanical Engineering and Electronic Information, China University of Geosciences (Wuhan), Wuhan 430074, China; 2 Queensland Micro- and Nanotechnology Centre, Griffith University, 170 Kessels Road, Brisbane, QLD 4111, Australia; sayhwa.tan@griffith.edu.au

**Keywords:** optofluidics, microfluidics, adaptive optics, optofluidic components, lab-on-chip applications

## Abstract

In the recent past, the field of optofluidics has thrived from the immense efforts of researchers from diverse communities. The concept of optofluidics combines optics and microfluidics to exploit novel properties and functionalities. In the very beginning, the unique properties of liquid, such as mobility, fungibility and deformability, initiated the motivation to develop optical elements or functions using fluid interfaces. Later on, the advancements of microelectromechanical system (MEMS) and microfluidic technologies enabled the realization of optofluidic components through the precise manipulation of fluids at microscale thus making it possible to streamline complex fabrication processes. The optofluidic system aims to fully integrate optical functions on a single chip instead of using external bulky optics, which can consequently lower the cost of system, downsize the system and make it promising for point-of-care diagnosis. This perspective gives an overview of the recent developments in the field of optofluidics. Firstly, the fundamental optofluidic components will be discussed and are categorized according to their basic working mechanisms, followed by the discussions on the functional instrumentations of the optofluidic components, as well as the current commercialization aspects of optofluidics. The paper concludes with the critical challenges that might hamper the transformation of optofluidic technologies from lab-based procedures to practical usages and commercialization.

## 1. The Origin of Optofluidics and Its Motivation

Optofluidics is referred to as the sciences and technologies which explore novel functions and the related physics through the marriage between light and fluid [1]. Optofluidics mainly aims at using microfluidic technologies to enable various optical functions or using optics to manipulate fluids at microscale. In recent years, growing interests and intense efforts have been directed to this field. Specifically, researchers have been developing reconfigurable optics using deformable fluids and in turn taking advantages of tunable optics to control or sense the fluid sample. The concept and implementation of using fluid to fabricate optical components and realize optical functions is not new. Stemming from the early 1900s, a deformable mirror was proposed and demonstrated by spinning fluid in a circular chamber to form a paraboloid shape [2]. The curvature of the mirror interface can be controlled by the spinning speed to construct a mirror with tunable focus. Another example of fluid-based optics is the commonly used oil-immersion microscope for enhanced magnification. These are good examples considered as rudimental development of adaptive optics. However, the most popular way to manufacture optics for centuries has been shaping and polishing solid materials, and the methodology to research and develop optofluidics has not been systematic until the recent maturity of MEMS and microfluidic technologies [3].

**Figure 1:**
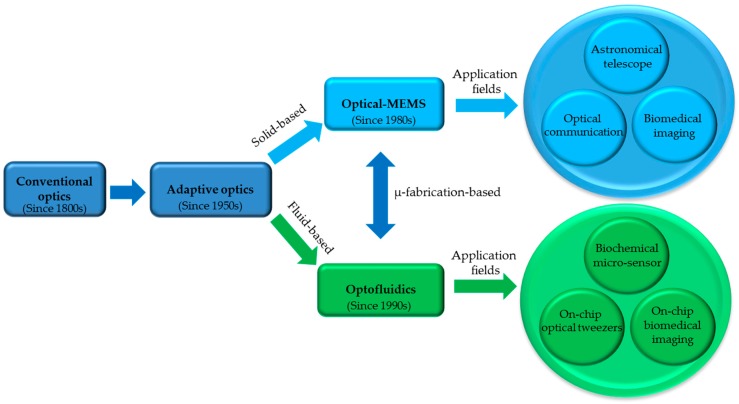
The evolvement of optofluidics from conventional optics with its applications in the fields of biochemistry, biomedical engineering, analytical chemistry, etc.

Figure 1 shows the evolution from conventional optics to tunable, adaptive and fluid-based optics. Since the first paper on light-steering using a micro-mirror published in 1980 by Petersen et al. [4], the MEMS technology has enabled the development of miniaturized solid-based optical devices mainly driven by the demands from digital display market, and subsequently helped to generate a new field-optical MEMS. Meanwhile, the advancements of MEMS technologies have also enabled the fabrication of microfluidic architectures with precisions down to nanoscale, including the fluid transfer in microchannels, the integrated mechatronic fluid control/monitoring units and fluid pumping/recycling sub-systems. Later on, the study and understanding of microfluidic mechanics opened up a platform to realize precise manipulation of fluid at sub-micron scale. All these developments and achievements have slowly paved the way to the point that it is feasible to fabricate and integrate the optical elements on microfluidic chips. This perspective will be mainly focused on the marriage between microfluidics and optics to develop tunable optical instruments and their applications for microfluidics. 

From a physical point of view, fluids can offer unique properties to realize novel optical functions: (1) replacing a liquid with another liquid inside an optical cavity can easily tune optical performance or function of the optical cavity; (2) refractive index (RI) gradients can be created by the control of diffusion between two miscible fluids; (3) an optically smooth interface can be formed between two immiscible liquids; and (4) light and sample can transfer along the same channel which allows long light–matter interaction length. These novel properties show promise to equip adaptive optical systems with more flexibilities and functionalities. In 2006, the review paper by Psaltis et al. summarized and categorized the early development of reconfigurable optical systems synergistically using optics and fluidics, and conceptualized the field of optofluidics [5]. Since then, optofluidics has intensively summoned research interests from the people working in multiple disciplines, such as biology, biomedical engineering, analytical chemistry, etc. 

A good number of review papers have been published with emphasis on a specific area dealing with applications using optofluidic technologies, such as whispering gallery mode sensor [6,7], label free bio-chemical sensor [8], microfluidic cytometry [9], energy production and harvesting [10], on-chip interferometry [11], on-chip treatment and manipulation of bio-cells [12,13], point-of-care genetic analysis [14], Raman spectroscopy [15], etc. There are also papers summarizing a group of specific optofluidic components, such as waveguides [16,17] and lenses [18,19], or discussing various optofluidic components operated by a specific working mechanism, for example fluid interface actuation [20]. However, few papers were dedicated to systematically discuss the development of fundamental optofluidic components and categorize them in terms of their working mechanisms. In this paper, we aim to comprehensively capture the miniaturized optical components using optofluidic technologies and classify them in terms of three working mechanisms: (1) liquid replacing; (2) refractive index gradient control; and (3) fluid interface deformation. Moreover, we discuss the basic optofluidic instrumentations by the utilization of those fundamental components. Finally, we look into the commercialization aspect of optofluidics and envision its future.

## 2. The Inventions of Optofluidic Components

**Figure 2:**
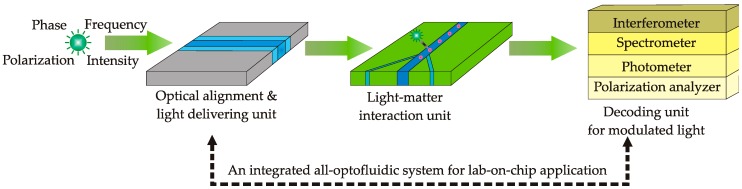
Schematic of an ideal all-optofluidic system for lab-on-chip applications.

Figure 2 illustrates the concept of an ideal optofluidic system with light emitter, light maneuvering/delivering channel, light–matter interaction cavity, and the decoding unit for the interpretation of the modulated light after interaction. One critical issue for the integrated optics is the feasibility and convenience of optical alignment. Different from free-space optics, which can be easily aligned by adjusting its physical positions, the integrated optics does not have that luxury of position adjustment after fabrication. Hence, self-tunability is of great importance for lab-on-a-chip optofluidic components. To manipulate and deliver the light with high precision and convenient alignment, a number of fundamental optofluidic components have been proposed and demonstrated. In the following, they will be discussed and categorized in terms of main three working mechanisms to achieve their tunability: (1) liquid replacing; (2) RI gradient control; and (3) fluid interface control.

### 2.1. Liquid Replacing

The function of most solid optical components is permanently fixed. Recently, the infiltration or injection of liquid into hollow structures has brought unique light–liquid interactions and excellent tunability of the optical components. Examples are infiltrated photonic crystal devices, such as photonic crystal fibers or planar photonic crystals. Thermal actuation [21], manual use of micropipette [22], laser-induced liquid recondensation [23] and soft lithography nanofluidic technique [24] have been utilized to inject liquid into photonic crystal structures. In porous photonic crystal devices, the selectively introduced liquids can significantly tune the transmission behavior of the device, such as emission wavelength [25], propagation direction [26] and speed [27] or spectral response [28,29].

Another example of replacing liquid inside an optical component is the optical switch based on the phenomenon of total internal reflection [30]. A mirror channel was filled with water (low refractive index) or salt solution (high refractive index) to determine whether total internal reflection happens or not, and therefore to allow whether the light beam can propagate through the mirror channel. Groisman et al. reported another optical switch using blazed diffractive grating [31]. For such a grating, diffraction maximum occurs when the optical path difference over one period of the grating is equal to an integer number of the wavelength of the incidence light. The fluid medium above the grating structure can be replaced by liquids with different RIs, and therefore the optical path difference can be changed accordingly to diffract the transmitted light beam with different propagation directions.

**Figure 3:**
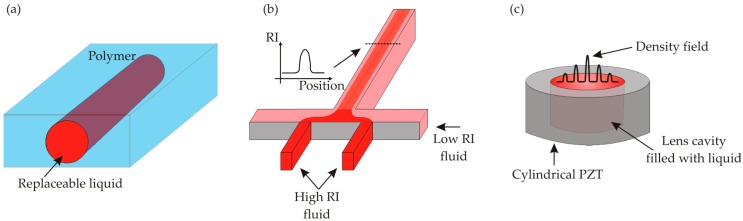
(**a**) A liquid core waveguide which can be tuned by replacing the liquid in the hollow channel; (**b**) an optofluidic GRIN lens configured by two miscible fluids; and (**c**) an acoustics-driven gradient refractive index lens.

Waveguide is one of the key elements in optical systems for confinement and transmission of light, equivalent to channel in terms of fluid transportation [16]. The integration and functionality of waveguide in microfluidic systems are of great interest in the research community. A tunable and reconfigurable waveguide can be used as sensors for biochemical analysis, which can offer longer light–matter interaction length and thus enhanced sensitivity [32]. A simple method to realize a tunable waveguide is to make a hollow channel filled with higher RI liquid embraced by lower RI solid material (Figure 3a) [33,34]. The optical characteristics can be easily adjusted by replacing the liquid inside the channel.

Llobera et al. reported a hollow prism built in a microfluidic system for optical sensing [35,36]. The analytes can be injected into the hollow prism chamber, and the interrogation light can be delivered by optical fiber inserted into the microfluidic platform. The RI shift of the analytes can be monitored by the measurement of deviation angle of the interrogation light. The sensing performance and efficiency were investigated using solutions of different concentrations of fluorescein. The devices were found to have good sensitivity in the measurement of absorption and shift of refractive index of the analytes.

### 2.2. Liquid RI Gradient Control

Instead of replacing the liquid inside an optical component, tuning refractive index of liquid by subjecting it to a flow field, temperature field, electrical field, acoustic field or mechanical strain are the alternative methods to manipulate the optical properties of light propagating in the optical material such as the optical path, phase change and polarization [37]. The generation of refractive index gradient is the most common method to manipulate light properties. The concept of refractive index gradient was already widely used in traditional fiber optics [38,39,40,41]. Light rays follow sinusoidal paths in the gradient-index fiber, with the advantage of decreasing the modal dispersion compared with multi-mode step-index fiber. Gradient refractive index (GRIN) also lends its strength to lens optics [42,43,44,45]. A proper distribution of index gradient in the direction perpendicular to the optical axis of the lens can help to eliminate the aberrations caused by traditional spherical lenses without varying the shape of the lens. The problem with solid gradient-index optics is the complete lack of tunability. The distribution of the gradient-index is fixed. Moreover, the fabrication process to establish such an index gradient is often complex, requiring field-assisted ion-exchange, micro-controlled dip-coating or vapor deposition.

The refractive index gradient can be controlled through the variation in concentration of a solution, which could be well defined by diffusion of microfluidic laminar flows. Based on this motivation, two-dimensional (in-plane) [46] and three-dimensional (out-of-plane) [47] optofluidic gradient refractive index lenses were proposed and demonstrated (Figure 3b). The laminar flow in microscale gives relative stability of optical performance to these devices, and it is found that the focal length of the lens can be tuned by the diffusion process, which can be well defined by setting a proper flow rate. Recently, Le et al. improved the in-plane gradient index lens by designing a micro-channel of cylindrical geometry with two-dimensional beam focusing capability, and carried out a parametric study to investigate the influences from mass fraction of core solution, flow rate and diffusion coefficient [48]. It is found that focal length can be effectively tuned by adjusting the mass fraction and the flow rate, and the beam spot size can be tuned by controlling the relative slip between the flows at core and cladding inlets. Another research shows that controlling the mass fraction can also be used to reduce aberrations of the gradient index lens [49]. 

Another mechanism to produce the refractive index gradient is by placing the liquid in a temperature gradient field [50]. In this work, a liquid core/liquid cladding configuration is employed to build an optofluidic waveguide. Two cladding streams at higher temperature sandwich a core stream at lower temperature, which creates a temperature gradient field. The refractive index and many other physical properties are temperature dependent. Therefore the temperature gradient field generates a distribution of refractive index with higher value at the center of the channel. This refractive index gradient enables the liquid core/liquid cladding flow a waveguide function. However, the thermo-optical coefficient *dn*/*dT* of most liquids is relatively small. Thus, a large temperature contrast is required to enable an effective waveguide function. Another drawback is the decay of temperature contrast along the flow direction due to dissipation of heat, which leads to unevenly distributed refractive index along the optical axis. This phenomenon causes a lot of optical energy loss during the propagation of light. To improve these drawbacks, Chen et al. proposed to integrate two heat sources into the lens-development chamber, and built up a high temperature gradient, which enabled strongly bending of light to realize lensing effect [51].

Acoustics as an alternative method to enable liquid GRIN lens can offer faster response than the temperature and diffusion driven mechanisms. It was found through theoretical studies and experimentations that acoustic standing wave created inside a cylindrical cavity by a circular piezoelectric transducer can generate a refractive index gradient and consequently result in lensing effect (illustrated in Figure 3c) [52,53]. In particular, the local refractive index of liquid was found to be dependent on a time-invariant density field superposed on the linear motion of the acoustic standing wave, and the time-invariant density field can be approximately described by a combination of Bessel functions. Later, generation and optimization of tunable Bessel beam was demonstrated using such acoustics-driven GRIN lens by McLeod et al. [54,55] Due to the convenience to incorporate the acoustics-driven GRIN lens into conventional optical systems, the tunable Bessel beam has quickly found its applications in bulk imaging [56], two-photon microscopy [57], three-dimensional microscopy [58], etc.

### 2.3. Fluid Interface Deformation

Recently, a number of optofluidic components based on fluid interface deformation have been proposed and demonstrated, including lenses, waveguides, prisms, apertures, etc. According to the light propagation direction relative to the plane of the substrate, which normally carries the test sample, those components can be divided into two categories: in-plane and out-of-plane types. The in-plane components manipulate beam shape in the plane of the substrate, whereas the out-of-plane ones manipulate the incident light perpendicular to the plane of the substrate.

**Figure 4:**
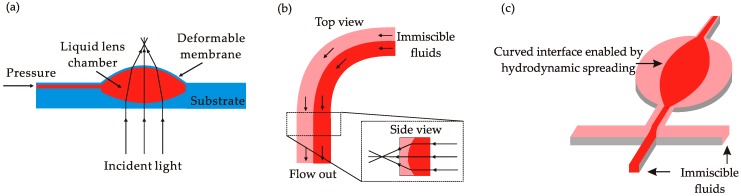
Illustration of optofluidics lenses: (**a**) out-of-plane lens tuned by applying pressure on a deformable membrane; (**b**) in-plane lens enabled by Dean flow; and (**c**) in-plane lens configured by liquid core liquid cladding structure.

A key advantage of the out-of-plane components is the compatibility with conventional solid-state optical system and thus the feasibility to replace the conventional ones with tunable optofluidic components in applications such as digital cameras or cell phones. Among the optofluidic components, optofluidic lenses have drawn considerable attention and have been intensively investigated due to their wide applications. A popular method to develop an out-of-plane optofluidic lens is to fill functioning fluid into a circular chamber sealed with a deformable membrane (Figure 4a). Inspired by a human eye’s crystalline lens, Ahn et al. proposed to use a deformable glass diaphragm with a thickness of 40 μm to enable a variable focal length [59]. However, the problem with this configuration is the high Young’s modulus of the glass diaphragm which limits the tuning range. Only up to 50 μm of deformation for a 10 mm lens can be achieved. To solve this problem, several groups proposed to use transparent elastic polymer (PDMS) as the diaphragm material to achieve a higher diaphragm deformation range [60,61,62]. Instead of using pneumatic tuning method to control the diaphragm, other working mechanisms such as acoustic [63], electromagnetic [64,65], electro-wetting [66] and electrochemical [67] actuations can be used to modulate the pressure in the liquid lens chamber and thus to tune the focus of the lens. Due to the inherently spherical shape, those lenses suffer from substantial spherical aberrations. Mishra et al. demonstrated to minimize the spherical aberration synthetically using electric field and hydrostatic pressure to manipulate the local curvature of the lens interface [68]. 

The advantage of in-plane components over out-of-plane ones is the possibility of integrating them into microfluidic networks which perform the sample analysis. A high level of integration helps to avoid the manual alignment of optical components as well as to reduce the cost of external bulky components. Different configurations of in-plane liquid/liquid or liquid/air interface optical components have been reported recently. They can be generally categorized into two groups: (a) interfacial intension based tuning; and (b) hydrodynamic tuning. The balance between the applied pressure and the interfacial tension can be used to tune the curvature of the liquid lens interface. Lien et al. fabricated a spherical micro-lens by injecting liquid-phase PDMS into the microfluidic channel [69]. One drawback of this fabrication method is the difficulty to pump highly viscous PDMS into a micro-channel and change the curvature of the PDMS–air interface within a short period of time. To improve this technique, several groups used liquids with low viscosity, such as water, to form the spherical interface driven by capillary force inside the microfluidic networks [70,71,72]. Since these naturally formed interfaces always present spherical shape, they can be only used for lens configuration.

Hydrodynamic tuning is a more flexible method to manipulate the liquid/liquid interface and realize different functional structures, such as waveguides, lenses, prisms, etc. In such configurations, multiple flows propagate in the channels with specific geometries. In a straight-line channel, a core flow with higher RI sandwiched by two cladding flows with lower RI can form a typical waveguide structure [73]. The width ratio between core and cladding flows can be changed by the pumping flow rates to tune the propagation mode of the light wave. By arranging the flows in a 90° curved channel (Figure 4b), the interface between the flows can bend along perpendicular direction to the substrate to realize a lensing function [74]. If the flow with a liquid core liquid cladding structure enters a shallow cavity (Figure 4c), the interfaces can deform according the geometry of the cavity boundary. Triangular [75,76], rectangular [77,78,79], hexagonal [80] and circular [81,82,83,84] cavities have been proposed to generate a curved interface which can bend the in-plane light according to the RI difference between the core and cladding flows and the shapes of the interfaces. Combining the concepts of liquid replacing and interface deformation, Song et al. designed a rectangular cavity with five inlet channels which allows the switching of fluids inside the cavity, and realization of a lens with both light converging and diverging capabilities [85].

**Figure 5:**
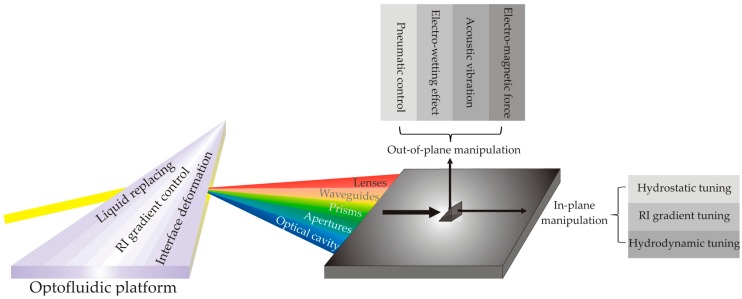
The categorization of optofluidic components in terms of their working mechanisms.

The categorization of optofluidic components is illustrated in Figure 5. In terms of working mechanisms, they can be divided into three groups: (1) liquid replacing; (2) RI gradient control; and (3) fluid interface deformation. Based on these working mechanisms, numerous components have been proposed and demonstrated, such as lenses, waveguides, prisms, apertures, optical cavities, etc. According to the direction of light manipulation, these components can be grouped as out-of-plane and in-plane components. The out-of-plane optofluidic components do not need to be necessarily confined in micro-channels, and thus they can be conveniently tuned by external forces generated by either separate devices or integrated functioning structures. For example, the droplet lens confined in a tube can be tuned by acoustic vibration from a microphone [63]; a liquid prism confined in a cylinder with the wall of the cylinder coated with metal electrodes can be tuned by electro-wetting effect [86]. However, for those in-plane components confined in microfluidic channels for lab-on-chip applications, using non-fluidic driven forces might require integration of complex functional structures, which needs complicated fabrication procedures, and therefore the entire cost of devices rises. To exploit fabrication-convenient and cost-effective solutions, several ways of tuning the optofluidic components have been intensively investigated, such as hydrostatic tuning, RI gradient tuning, and hydrodynamic tuning. Owing to the promising future of optofluidics for lab-on-chip applications, the following section will be more focused on the discussion of using in-plane optofluidic components to develop the optical instruments for bio-chemical analysis and treatment. 

## 3. Instrumentation of Optofluidics

Based on the abovementioned optofluidic components, a variety of detection and analytical tools have been developed, which have applications in biomedical research, biochemistry, pharmaceuticals, healthcare, and environmental monitoring. The integration of optofluidic components on a microchip can greatly reduce the fabrication cost and significantly downsize the entire systems. It is promising and possible to make the system portable for field test even in remote regions that cannot afford high-cost equipment. 

Fundamentally, light carries the information encoded with its basic properties, such as phase, electromagnetic frequency, intensity or polarization. The interaction of light with the inspected sample can modulate its properties, which need to be decoded by specific tools such as interferometer, spectrometer, photometer and polarization analyzer (Figure 2). This section will discuss the instrumentation of optofluidics under such categories.

### 3.1. Optofluidic Interferometry

**Figure 6:**
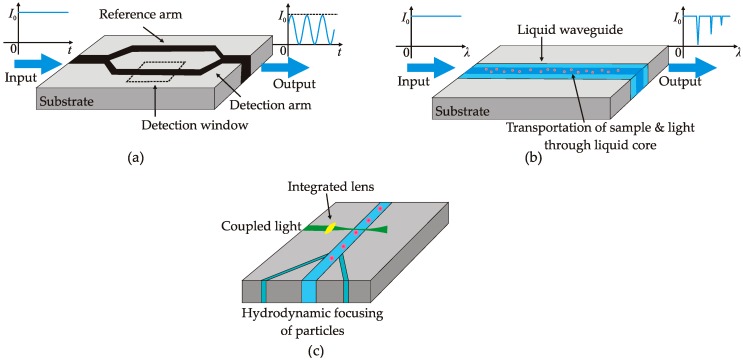
Schematics of functional optofluidic devices: (**a**) integrated Mach–Zehnder interferometer for label-free detection of biochemical sample; (**b**) optofluidic enhanced Raman spectroscopy for analytical chemistry; and (**c**) a typical configuration for optical detection or manipulation of micro-particles.

Thanks to the realization of on-chip waveguide, the Mach–Zehnder interferometer (MZI) can be easily developed with both sensing and reference arms aligned on an optofluidic chip (Figure 6a). The sensing arm passes through a detection window with its evanescent field penetrating into the fluid analyte in the detection window. Any subtle RI variation in the analyte can result in a change in the optical path. The change in optical path changes the intensity from the output end. By measuring the signal change, the MZI can precisely measure the phase difference between the light waves propagating through the two arms.

The first on-chip MZI was realized by growing Si_3_N_4_ on a silicon substrate to configure the two waveguides arms, and successfully measure the concentration of biomolecules [87,88,89]. Following works involved the improvements in the waveguide material and configuration of the interferometer [90]. It has been demonstrated that the environmental noise can be canceled by proper design of the reference arm [91]. To improve the precision in signal measurement, a phase modulator can be incorporated in the reference arm to track the quadrature points and enable linearity in signal changing [92]. However, the transduction signal of those configurations relies on the interaction between evanescent field of guided mode and the analyte, which requires sufficiently long interaction length in order to achieve a decent sensitivity. To improve the sensitivity, an extrinsic configuration with the sensing arm orthogonally crossing the analyte flowing channel has been proposed to increase the light-analyte interaction length [93,94]. The drawback of this configuration is that the separation between the two waveguides across the detection window might cause severe transmission loss. Lapsley et al. improved the light coupling efficiency by incorporating two polymer lenses to collimate divergent beams on their chip [95]. Another method to enhance the sensitivity is to couple the light into a liquid core waveguide as the sensing arm [96,97,98]. The light and the sample fluid can transfer along the same channel to achieve a maximum light–matter interaction length.

### 3.2. Optofluidic Spectroscopy

Spectroscopy, as an important optical tool, can measure analytes at very low concentration level based on the absorption, fluorescence or Raman characteristics. The main limitation to integrate the spectroscopic function on a micro-chip is the short light–matter interaction length due to the inherently small size of the chip. However, the invention of the optofluidic waveguide enables an enhanced light–matter interaction to achieve maximum sensitivity by offering a way to transmit the light and analytes along the same guide (Figure 6b).

In the work of Yin et al. [99], an anti-resonant reflecting optical waveguide (ARROW) [100] was used to carry the analyte in the hollow liquid core. The excitation of fluorescence was implemented by coupling an external laser into the liquid core with the fluorescent molecules. The fluorescence signal can be carried by the propagation mode and transmitted through the ARROWs with enhanced interaction and collected by a photomultiplier tube. To improve the detection limit (DL), the same group proposed a configuration with the excitation waveguide perpendicularly aligned to fluorescence waveguide [101]. Single molecule detection can be achieved on a planar microfluidic network. Testa et al. proposed a configuration with the fluidic part fabricated with polymer which is stacked on an ARROW structure fabricated with silicone [102]. This configuration has the flexibility to replace the fluidic module with specific functions. The concept of using liquid core waveguide has also been demonstrated to measure the refractive index of the analyte [103] and the concentration of bio-molecules [104,105] with extremely high sensitivity by looking at the spectral shift of the transmitted light. Besides the waveguide, the optofluidic prism [35,36] and grating [106] can also find their applications in sensing the variation of RI of the analytes.

### 3.3. Optofluidic Cytometry

A traditional flow cytometer employs bulky optics to inspect the flowing particles of micro-size or biological cells aligned in a single line. The scattered light or fluorescence signal can be collected and analyzed to reveal the physical and chemical properties of the particles or cells. Since both delivery and collection of light involve external bulky lenses as well as the alignment of optics, the system can hardly be used for field test.

Recently, many efforts have been dedicated to downsize the cytometry system and make it portable. The microfluidic platform offers an efficient way to align the particles along a single streamline due to the laminar flow characteristic. The most common implementation is to utilize a flow carrying particles sandwiched by two sheath flows. The hydrodynamic focusing effect allows the particles following a line to pass through the inspection window [107] (Figure 6c). The inspection light can be simply delivered via external objective lens and the scattered light from the particles, which can be described by Mie theory, can be collected from either backscattering direction or forward-scattering direction. To further downsize the system, external optics needs to be integrated on the same microfluidic system. Optical fiber can be used for both light delivering and collecting [108,109]. However, the divergent beam at the output end of the fiber leads to inaccuracy of detection due to the enlarged inspection region. Several groups proposed to integrate a micro-lens by direct shaping of PDMS–air/liquid interface to focus the light beam delivered from an optical fiber [110,111,112]. The focused beam crossing the inspection window enables the detection of single particle and prevents the misinterpretation of the collected signal. Further improvement involves the use of an optofluidic lens configured by hydrodynamic spreading to focus the beam without any light scattering which might be caused by the lens with solid-air interface [78,113].

### 3.4. On-Chip Manipulation of Micro-Object

The manipulation of micro-object involves trapping, sorting, and separation, which can find their applications in the fields of chemistry, biology and colloidal science. The optical method, which is referred to as optical tweezers [114,115], has played an important role due to its accuracy and flexibility to handle single or multiple micro-objects. 

Combining laminar flow characteristics in a microfluidic channel with optical trapping, sorting of particles or cells can also be achieved. The absence of turbulence in a microchannel gives the particle or cells a predictable drag force along flow direction. The optical trap will exert a gradient force along a defined direction, which combined with drag force results in a net force that can redirect particles from their original tracks into the designated fluidic branch [116,117]. Similar work was done by Applegate et al., who used laser diode bars and focused the beam in a microchannel to achieve particle sorting [118]. With such a configuration, bovine red blood cells can be captured by the optical trap with linear shape, which was placed at an angle to the flow direction, and the cells were conveyed along it before being released at its end, thereby sorting cells according their sizes.

Instead of using external optical instruments to manipulate the light, the evanescent field trapping is another type of optical method to manipulate particles which can employ on-chip waveguide to generate an intensity gradient field of light. Schmidt et al. investigated the forces exerted by the evanescent field, which can attract the particles to the surface of the waveguide and at the same time propel the particles along the propagation direction of light [93]. Particles with a diameter of 3 μm and velocity up to 28 μm/s were successfully sorted by their device. Besides the gradient force, the scattering force of light can also be used for on-chip particle manipulation. Both free space optics [119,120] and embedded fiber [121] have been demonstrated to deliver the scattering force on the flowing particles confined in a microfluidic environment and divert them to a new routing. 

## 4. Commercialization of Optofluidics

Leveraging the superior reconfigurable properties, optofluidic systems can be dynamically tuned without any moving mechanical parts, which can be utilized to develop faster and more compact optical systems. Thus far, several concepts have been commercialized with their applications in a variety of fields. Due to the compatibility with conventional optical systems, the out-of-plane optofluidic configurations, especially lenses, were the forerunners during the development of optofluidic products. An acoustics-driven GRIN lens (TAG Optics Inc., Princeton, NJ, USA [122]) is available on market with its applications in machine-vision, biological microscopy and laser material processing. The technology relies on changing the refractive index of liquid confined in a cylindrical chamber by acoustic pressure waves. Since the wave travels at sound speed, the redistribution of RI gradient can respond within 10–30 μs. Electro-wetting based liquid lenses were commercialized by Varioptic [123]. Besides the imaging functions, the lens of this type possesses the capability of focusing and steering laser beam in *X*, *Y* and *Z* axis by selectively controlling the electrodes, which can find its application in ophthalmology. Other actuation methods, such as using deformable diaphragm and electro-active polymer, were also used in the production of tunable liquid lenses by Optotune [124].

For the lab-on-chip applications, the in-plane waveguides are commonly used to develop fluid sensor and particle tweezers due to the convenient fabrication and control. World Precision Instruments commercialized a device using liquid core waveguide for the measurement of light absorbance of analytes [125]. A NanoTweezer based on the evanescent-field trapping using integrated waveguide was developed by Optofluidics [126]. However the overall sizes of these devices are still bulky, since they require external optics, in particular lenses, to perform further analysis. Unlike the out-of-plane optofluidic lenses, the in-plane lenses are still in their early research stage. Several reasons impede the on-chip optofluidic in-plane lenses from their integration into commercial products. In terms of the hydrodynamically developed in-plane lenses, they consume liquid when at working state, and, to date, no technology has been demonstrated to recycle and reuse the functioning fluids for this type of lens. The printed polymer in-plane lens does not consume liquid in principle, but its surface quality depends on the fabrication process. Furthermore, the focal length of the printed lens is fixed after fabrication, on-chip optical alignment during the test is impossible. The capillary effect of liquid in micro-channels can be used to form lens interface without continuously consuming liquids, and the curvature of the interface can be tuned by balancing surface tension and other driving forces. Thus far, the fastest way to tune the liquid lens in micro-channels is by the control of pneumatic pressure [70]. However, the response is still slow when compared to sound or electro-wetting driven out-of-plane liquid lenses, and sensitive pressure controller might be needed to balance the surface tension of liquid in microscale, which will increase the entire cost of the system. Those might be the reasons that the currently demonstrated in-plane optofluidic lenses have not been widely utilized for commercial products. 

Recently, several ways have been explored to manipulate the fluid interface at microscale with integrated control units in the field of droplet-based microfluidics. Thermal heating generated by integrated microheater has been demonstrated to effectively change the interfacial tension between two immiscible fluids [127,128]. Maxwell stress induced by integrated electrodes can also be used to change the interfacial tension [129,130]. The manipulation of interfacial tension can dynamically adjust the size and shape of micro-droplets which essentially can serve as tunable micro-lens. The concept of the out-of-plane acoustics-driven lens can also be leveraged to realize in-plane tunable GRIN lens by imposing surface acoustic wave (SAW) to generate gradient density field using affordable and self-aligned interdigitated transducers [131]. The authors believe all these methods can be leveraged to develop cost-effective in-plane optofluidic lenses with high tuning response and precise manipulation, which would benefit the further commercialization of on-chip optofluidic systems. 

## 5. Discussions and Conclusions

This paper gives an overview of the developments of fundamental optical elements using fluid referred as optofluidic components. Driven by the demands of lab-on-chip applications, many efforts have been devoted to realize the integration of optofluidic components into microfluidic systems. With the advancements of MEMS technologies, a variety of optofluidic components can be miniaturized and printed on the same planar structure with other assay modules. Those optofluidic components can be categorized based on their working mechanisms: (1) liquid replacing; (2) RI gradient control; and (3) liquid/fluid interface deformation. Owing to the reconfigurable features, these optofluidic components possess a high level of tuneability to make the optical system highly adaptive. Adaptability as well as the miniaturization can significantly downsize the entire system and make the system portable for field test. Instead of using bulk optics in free space, the integrated optics can greatly reduce the cost of system and thus hold a promise for affordable point-of-care diagnosis even in those regions suffering from poverty.

Based on the developments of fundamental optofluidic components, many of the optical tools for biochemical analysis and treatment can be realized with miniaturized and simplified architectures. The invention of optofluidic waveguide enables an efficient way to print the optical arms of an interferometer on a planar structure with the sensing arm directly interacting with the analytes; the liquid core waveguide allows the transfer of sample fluid and light along the same guide. The enhanced interaction between light and fluid enables integration of spectroscopy into microfluidic platforms; the developments of optofluidic lenses enable enhanced detection of micro-particles or even single fluorescent molecule without using external bulky optics. It is envisioned that an all-optofluidic system can be practically realized with all optical functions integrated into a single chip in the near future.

To achieve this goal, several critical challenges need to be addressed carefully. Firstly, many of the optofluidic components consume functioning fluids even when working at steady state. An effective way needs to be figured out to recycle and reuse the fluid, which can further lower the operational cost of the system and make the system more practical. The stability of fluid pumping is another critical concern. Any discontinuity in pumping might introduce disturbance to the functioning of optofluidic components, which can result in inaccuracy of the analysis or malfunction of the optical treatment. Besides the discontinuity in pumping, the vibrational noise from the environment may also introduce perturbation to the optofluidic system. The environmental noise can be isolated for the free-space optics by mounting the optical elements on an anti-vibration table, but it is not practical to do the same for the optofluidic systems since they are born to be used for field test and point-of-diagnosis. Hence, cost-effective housing of on-chip optofluidic systems with effective anti-noise techniques has to be developed for the practical use and their further commercialization. Different from the manipulation of light in free space using glass-based optical elements, it is difficult to tightly focus a light beam with a high numerical aperture using optofluidic components, since most structural materials for microfluidic system have very high refractive index around 1.4 and the RI of liquids lies between 1.3 and 1.6 RIU. This drawback of optofluidic components may hamper the developments of optical tweezing with high strength and high-resolution imaging. Efforts need to be dedicated to exploit novel fabrication methods or materials for the light manipulation using on-chip elements.
